# Predictors of problem-solving skills among emergency medical services staff in Iran: A cross-sectional correlational study

**DOI:** 10.3389/fpsyg.2022.934569

**Published:** 2022-07-27

**Authors:** Masoud Saeedyan, Mohammad Ali Mohammadi, Alireza Mirzaei, Naser Mozaffari

**Affiliations:** ^1^Department of Medical-Surgical Nursing, School of Nursing and Midwifery, Ardabil University of Medical Sciences, Ardabil, Iran; ^2^Department of Emergency nursing, School of Nursing and Midwifery, Ardabil university of medical sciences, Ardabil, Iran; ^3^Department of Nursing, School of Nursing and Midwifery, Ardabil University of Medical Sciences, Ardabil, Iran

**Keywords:** emotion regulation, emergency medical services, Iran, problem solving, response time, staff

## Abstract

**Background and aims:**

Pre-hospital emergency technicians face many problems in the workplace daily, so the ability to solve or overcome them in the workplace is essential. This article aimed to assess the predictors of problem-solving skills among emergency medical services staff in Iran.

**Methods:**

This study was cross-sectional correlational research. Using convenience sampling methods, 140 emergency medical services (EMS) staff were enrolled in the study. Response time was assessed using ASAYAR software, problem-solving skills (PSS) were measured using the Hepner Petersen Problem Solving Questionnaire (PSI), and cognitive emotion regulation strategies were assessed using the Garnfsky Cognitive Emotion Regulation Questionnaire. Descriptive statistics, *t*-test, one-way analysis of variance (ANOVA), Pearson's *r* correlation coefficient, and multiple linear regression analysis were applied using SPSS 14.0.

**Results:**

The results of our study showed that the total mean score for problem-solving skills was 136.84 (14.65) (range, 175–107 points). Multiple linear regression indicated that refocusing on planning, positive evaluation, stress management courses, delays and their causes, positive refocusing, catastrophizing, and acceptance were effective predictors of problem-solving skills in emergency personnel, accounting for 54% of the total variances.

**Conclusion:**

This study is one of the first studies in this field. Based on our findings, individuals who consider their emotions and feelings when solving problems are better able to use the process of thinking and problem-solving skills. Therefore, by training people in the field of emotional regulation skills, the skills to solve problems technicians can be increased.

## Introduction

Nowadays, providing public health services in a country is highly important. These healthcare services are considered an essential part of the pre-hospital emergency system (Lee et al., 2018). Pre-hospital emergencies in Iran dates back to 1975 when the first emergency care unit was established in the capital, Tehran, after which pre-hospital emergency departments, including urban and road centers, were gradually established in other provinces of Iran (Bijani et al., 2021). The Emergency Medical Services (EMS) personnel have an associate's or bachelor's degree in anesthesiology, operating room, and emergency medicine, or a bachelor's or master's degree in nursing (Habibi Soola et al., 2022; Mirzaei et al., 2022). The EMS personnel are often the first people to be exposed to numerous emergencies, from heavy vehicle crashes and natural disasters to minor injuries and illnesses (Bijani et al., 2021). If emergency services are provided promptly and completely, the mortality rate and disabilities caused by some diseases and accidents will be reduced (Garner et al., 2018).

In pre-hospital emergency care, time frames (seconds and minutes) can mean the difference between life and death or between severe disability and having a normal life (Bijani et al., 2021). Response time is of great importance in pre-hospital emergency care and is considered the most important indicator to control the efficiency of a pre-hospital emergency system (Alnemer et al., 2016; Lee et al., 2018). Studies have shown that a 1 min increase in response time may lead to a decrease in patient survival (Goto et al., 2018). Tim et al. showed that the average response time is much higher than the golden time (Timm et al., 2014), which is ideally considered in different countries to be between 4 and 20 min (Zeraatchi et al., 2018). According to the regulations of the Iranian Ministry of Health, the response times in pre-hospital care in Iranian cities and roads are 8 and 15 min, respectively (Saburie et al., 2017; Taheri et al., 2017). The EMS technician is one of the most important factors involved in reducing or increasing this response time (Cone, 2021). In choosing the EMS technicians, various factors such as personality traits, values, interests, skills, family circumstances, and social conditions should be considered (SafarAbadi et al., 2015). In other words, they must be able to accurately diagnose problems and choose the best solutions in the shortest possible time because they constantly face various professional and stressful problems during the shift and must make the best decision in the fastest time. There are various ways to deal with these problems, including problem-solving and emotion management skills (Huang and Flores, 2011).

Emotion management skills refer to the cognitive emotion regulation by which people can properly moderate their feelings and emotions (Ashori and Najafi, 2021). Given the positive association between cognitive emotion regulation and increased self-confidence as well as the emergence of better social behaviors, it is not surprising that this multidimensional structure- adaptive or maladaptive emotion regulation strategies-affects cognitive problem-solving skills embedded in decision-making and critical thinking ability (Aminabadi et al., 2011; Te Brinke et al., 2018). Problem-solving skills include a combination of behavioral, cognitive, and emotional reactions that manifest when a person encounters everyday problems to adapt to intrinsic actions and overcome the pressures arising from them (Ancel, 2016; Bayram et al., 2022). Accordingly, they try to search for various sources and then apply useful strategies (Huang and Flores, 2011; Tan et al., 2019). Problem-solving skills include the three components of self-confidence in problem-solving, avoidance approach, and personal control, which indicate a person's belief in the ability to solve problems, their willingness to ignore problems, or confrontation with problems, and using different strategies to control behavior and purposefulness in the problem-solving process, respectively (Feizi Konjini et al., 2016). Studies have shown that when a person encounters stressful problems and events, problem-solving skills and emotion management skills become more important and help solve the problems easier and faster (Bahtiyar and Can, 2016; Bayram et al., 2022). Thus, having problem-solving skills is a requirement for EMS technicians. Further, because of their job sensitivity, the EMS staff must be able to think critically to analyze, prioritize, and reorganize emergencies (Heidari and Shahbazi, 2016; Tan et al., 2019). One of the challenging situations that can undermine problem-solving skills or make decision-making difficult is emotion regulation in confrontation with critical situations. The results of studies also show the relationship between problem-solving skills and emotion regulation ability (Madanifard et al., 2016).

Pre-hospital emergency services play a key role in reducing deaths and complications due to injuries and diseases. There is also a need for regular performance monitoring, especially in indices affecting the process of providing services to patients, including human resources, using problem-solving skills. Moreover, studies in problem-solving areas in the pre-hospital emergency system are scarce. Hence, this study was conducted to investigate the predictors of problem-solving skills among emergency medical services staff in Iran.

## Materials and methods

The research population of this descriptive-analytical study comprised EMS technicians working in the emergency medical centers of Ardabil city (*n* = 160). The inclusion criteria consisted of more than 6 months of clinical experience in the pre-hospital emergency, willingness to participate in the study, complete missions performed in the base area, and registered in the ASAYAR system. The exclusion criterion was canceled for off-base missions. After observing ethical considerations and obtaining the necessary approval, the researchers obtained the list of the personnel of urban and road EMS bases by a convenience sampling method. A total of 160 people met the inclusion criteria. The questionnaire was distributed among them, and 140 questionnaires were eventually returned. In this study, the effective recovery rate of the questionnaire was 87% (140/160). For each technician, the demographic characteristics, problem-solving skills, and emotion regulation questionnaires, as well as response time information in 1 month (July 1 to 31, 2019) were completed, from which a total of 3,000 missions were completed. Information was collected from July to 1 October 2019.

## Demographic forme

The demographic characteristics included age, work experience, marital status, technician's position, number of children, employment status, level of education, field of study, having a delay and the reasons for it, and passing stress management, problem-solving, and time management courses.

## ASAYAR software

This system uses the Global Positioning System (GPS footnote) to record the response time of senior technicians that decide on emergency missions from the time of the emergency call to the scene of the accident or the patient's bed.

## Problem solving inventory (PSI)

The Problem Solving Inventory (PSI) is a 35-item tool developed by Hepner and Peterson (Heppner and Petersen, 1982) that measures a person's perceptions of problem-solving abilities and problem-solving style in everyday life based on a Likert scale, ranging from strongly agree (score 6) to strongly disagree (score 1). The PSI has three dimensions of trust in problem-solving with 11 items, avoidance approach with 16 items, and personal control with 5 items. Some items (9, 22, and 29) are not scored. Questions 29, 30, 32, 34, 25, 21, 26, 15, 14, 9, 11, 4, 3, 2, and 1 are negative propositions and have the opposite score (Sahin et al., 1993). A total score can be calculated as a general index of problem-solving appraisal that ranges from 32 to 192 (Sahin et al., 1993). No cut-off point was defined for the evaluation of the scale. Lower scores on each factor and on the total PSI score are considered more functional. The reliability of the instrument in the study of Hepner and Petersen was reported to be 90% by Cronbach's alpha (Heppner and Petersen, 1982). This rate was 83% in the study of Yaghoobi and Motevalli (2020). In the present study, the reliability of the instrument using Cronbach's alpha was 77%.

## Cognitive emotion regulation questionnaire (CERQ)

The CERQ, developed by Garnefski and Krraij (Garnefski and Kraaij, 2006), is an 18-item questionnaire that measures cognitive emotion regulation strategies in response to life-threatening and stressful events on a Likert scale ranging from 1 (never) to 5 (always). This questionnaire includes 9 subscales of self-blame, other-blame, rumination, catastrophizing, putting into perspective, positive refocusing, positive reappraisal, acceptance, and planning (Garnefski and Kraaij, 2006). Cognitive emotion regulation strategies in this questionnaire are divided into two general categories, namely, adaptive strategies (adapted) and non-adaptive strategies (non-adapted). Adaptive strategies include positive refocusing, positive reappraisal, acceptance, and refocusing on planning. Maladaptive strategies consist of self-blame, other-blame, rumination, and catastrophizing. The reliability of the questionnaire in Garnefsky and Kraaij's research for the 9 mentioned subscales was reported to be between 62 and 80% (Garnefski and Kraaij, 2006). The reliability of this questionnaire in Hassani's study was between 68 and 82% (Hasani, 2010). In this study, Cronbach's alpha was 76% for positive strategies, 77% for negative strategies, and 0.78 for the whole questionnaire.

## Data analysis

SPSS 14 was used for data analysis. Frequency, percentage, mean, and standard deviation were used to describe the participants' characteristics, response time, problem-solving skills, and emotion regulation levels. A Pearson correlation was used to test the associations between the studied variables (problem-solving skills and emotional regulations) and multivariate linear regression tests were used to identify the predictors of problem-solving skills. A significance level of 0.05 was considered.

## Ethical considerations

Before data collection, ethical approval was obtained from the Research Committee of Ardabil University of Medical Sciences (approval number: IR.ARUMS.REC.1398.228). The researchers were allowed to access the study site and collect data. All participants expressed their consent after being informed of the research objectives and method. This study adhered to the principles of the 2013 Helsinki Declaration. Participation was voluntary and informed written consent was obtained from each respondent. To ensure anonymity and confidentiality, no personal identifier of the respondent was used in the collected data.

## Results

The overall response rate in this study was 87%. Their mean age was 32.99 (SD: 7.96), and their mean clinical experience was 8.1 years (SD: 7.16). The majority of the participants (89%) were married, 35.71% were officially employed, and 71.80% had a bachelor's degree. The majority of samples (90.70%) had not passed the problem-solving and time management courses, and 80.7% did not have a delay in mission. Other demographic characteristics are shown in Table 1.

**Table 1:** General characteristics of emergency medical services staff (*N* = 140).

**variables**		***N*** **(%)**	**Mean (SD)**
Age			32.99 (7.96)
Work experience			8.1 (7.16)
Marital status	Single	51 (36.4)	
	Married	89 (63.6)	
Position	EMT-Intermediate	57 (71.40)	
	EMT-Paramedic	83 (28.59)	
Number of children	No	81 (57.9)	
	1–3	59 (42.1)	
Type of employment	official	50 (35.71)	
	contract	32 (22.58)	
	Contractual	12 (8.56)	
	Projective	46 (33.15)	
Educational level	Associate	25 (17.58)	
	Bachelor's and master's	115 (82.42)	
Reasons for the delay	There was no delay	113 (80.7)	
	Fatigue	4 (2.9)	
	A few cases	10 (7.1)	
	others	13 (9.3)	
Stress management course	Yes	126 (90)	
	No	13 (10)	
Participate in a problem-solving course	Yes	13 (9.3)	
	No	127 (90.7)	
Participate in time management courses	Yes	13 (9.3)	
	No	127 (90.7)	
Field of Study	Nursing	82 (58.6)	
	Anesthesia	15 (10.7)	
	surgery room	6 (4.3)	
	Medical emergency	28 (20)	
	Relief and Rescue	9 (6.4)	

The distribution of mean scores of problem-solving skills and its sub-scales showed a mean score of 48.12 (5.86) (range: 29–63) for PSC, 65.87 (7.48) (range: 49–65) for CA, and 19.25(4.78) (range: 8–30) for PC. The overall mean PSI was 136.84(14.65) (range: 107–175). The EMS personnel mostly used positive strategies to regulate emotions in stressful situations. However, the lowest mean score among emotion regulation components was related to the total score of negative strategies and other-blame subscale (Table 2).

**Table 2:** Descriptive statistics of the study variables (*N* = 140).

**Variable**	**Mean**	**SD**	**Min**	**Max**
Total PSI score	136.84	14.65	107.00	175.00
Problem solving confidence	48.12	5.86	29.00	63.00
Approach avoidance style	65.87	7.48	49.00	85.00
Personal control	19.25	4.78	8.00	30.00
Positive strategy	3.54	0.51	1.80	4.70
Acceptance	6.71	1.90	2.00	10.00
Positive refocusing	6.06	2.02	2.00	10.00
Refocusing planning	8.20	1.80	2.00	10.00
Positive appraisal	7.78	1.73	3.00	11.00
Putting in to perspective	6.72	1.69	2.00	10.00
Negative strategy	2.65	0.68	1.13	4.25
Self-blame	4.95	2.31	2.00	10.00
Rumination	7.03	1.85	2.00	10.00
Catastrophizing	4.88	1.70	2.00	9.00
Blame others	4.34	1.86	2.00	9.00

The results showed that the total response time of technicians (roads and cities) was 10.49(2.79) minutes (Table 3). The time spent taking patient histories (T1) and the overall response time in cities were statistically longer, i.e., weaker than their defined standards. However, the total response time on the road was significantly less (better) than its defined standards. In addition, the response time was better in Ardabil City than in other cities in Ardabil Province.

**Table 3:** Mean score response time of participating technicians after receiving the mission message.

**Item**	**Time (minutes)**
Time T1	1.21 (0.26)
Response time in the city	10.22 (2.45)
Response time on the road	10.57 (3.36)
Response time in Ardabil	10.21 (2.63)
Response time in counties of Ardabil province	11.41 (3.99)
Total response time (roads and cities)	10.49 (2.79)

Table 4 shows the relationship between problem-solving skills and their sub-scales and the positive and negative strategies of EMS personnel. Problem-solving skills were significantly inversely correlated with negative strategies (*r* = −0.370, *p* < 0.001), but they were not correlated with positive strategies. Self-confidence in problem-solving had a significantly negative correlation with negative strategies (*r* = −0.198, *P* < 0.05) and a significantly positive correlation with positive strategies (*r* = 0.344, *p* < 0.001). The rest of the results for correlation between the variables are presented in the table.

**Table 4:** Correlations among the study variables (*N* = 140).

**Variable**	**1**	**2**	**3**	**4**	**5**	**6**
Total problem-solving inventory score	1	0.778^**^	0.897^**^	0.758^**^	0.133	−0.370^**^
Problem solving confidence	0.778^**^	1	0.529^**^	0.368^**^	0.344^**^	−0.198^*^
Approach avoidance style	0.897^**^	0.529^**^	1	0.613^**^	−0.040	−0.333^**^
Personal control	0.758^**^	0.368^**^	0.613^**^	1	0.046^**^	−0.428^**^
Positive strategies	0.133	0.344^**^	−0.040	0.046	1	0.131
Negative strategies	−0.370^**^	−0.198^*^	−0.333^**^	−0.428^**^	0.131	1

*1, Total Problem-Solving Inventory score; 2, Problem Solving Confidence; 3, Approach Avoidance Style; 4, Personal Control; 5, Positive strategy; 6 Negative strategy*.

* *P < 0.05*,

** *P < 0.01*.

Table 5 indicates the results of multiple regression analysis regarding the predictors of problem-solving skills. A multiple linear regression analysis was performed using problem-solving skills as dependent variables and emotion regulation and demographic characteristics as independent variables. The Durbin–Watson test statistic was 1.88, thereby falling within 1.5–2.5; as such, there was no linear relation in the data. The variance inflation factor ranged from 1.04 to 4.26, whereas tolerance ranged from 0.23 to 0.95 with no multicollinearity among independent variables. Of these 16 variables, 7 variables were identified as significant predictors of problem-solving skills. The coefficient of determination (*R*^2^) of the regression model showed that 54% of the total score of problem-solving skills can be explained by the input variables of the model. Among the variables fed into the model using the ENTER method, reported in the table, the refocusing on planning, positive reappraisal, passing the stress management course, having delays and their reasons, positive refocusing, and acceptance variables were predictors affecting problem-solving skills among EMS personnel.

**Table 5:** Multiple regression analysis predicting problem-solving skill.

**Variables**	***B***	**Beta**	***t***	***P*** **-value**	**95% confidence interval for** ***B***
(Constant)	142.353		8.196	<0.0001	107.974–176.731
Response. Time	−0.435	−0.083	−1.329	0.186	−1.083–0.213
Self-blame	−0.650	−0.103	−1.449	0.150	−1.539–0.238
Acceptance	−1.606	−0.209	−3.123	0.002	−2.623 to −0.588
Rumination	0.508	0.064	0.811	0.419	−0.732–1.748
Positive refocusing	−1.498	−0.207	−2.869	0.005	−2.532 to −0.465
Refocus on planning	2.687	0.330	4.616	<0.0001	1.535–3.839
Positive reappraisal	2.739	0.324	4.864	<0.0001	1.624–3.853
Putting into perspective	0.565	0.066	0.908	0.366	−0.667–1.798
Catastrophizing	−1.531	−0.178	−2.210	0.029	−2.902 to −0.160
Blaming others	−1.025	−0.130	−1.647	0.102	−2.257–0.207
Age	0.231	0.126	1.061	0.291	−0.200–0.662
Number of children	−3.156	−0.198	−1.769	0.079	−6.687–0.375
Position	2.660	0.089	1.308	0.193	−1.364–6.684
Participate problem-solving course	−12.196	−0.242	−1.922	0.057	−24.753–0.362
Stress management course	−13.736	−0.312	−2.515	0.013	−24.546 to −2.926
Reasons for the delay	−0.866	−0.255	−3.724	<0.0001	−1.326 to −0.405

*R^2^, 0.541; Adjusted R^2^, 0.481; F _(9.057)_, P < 0.001 A Dependent variable: problem-solving skill*.

## Discussion

Problem-solving skills in medical emergencies refer to the ability to quickly solve health problems through critical thinking based on knowledge and experience (Yang and Kim, 2022). Problem-solving skills require confidence, avoidance approach, and control. Self-confidence has a positive effect on the problem-solving process and outcome. The avoidance approach refers to the hardworking approach to avoiding bad things and is similar to stress coping. In the problem-solving process, it is necessary to have control over the individual's awareness and behavior (Arble and Arnetz, 2017). This study aimed to investigate problem-solving skills and their relationship with emotion regulation and response time among emergency medical services staff in Iran.

EMS staff had poor problem-solving skills, which is consistent with the results of previous studies (Yavuz et al., 2010; Avsarolu, 2019). Further, the results of Altun and Uslu showed that the participants had better problem-solving skills (Altun, 2003; Uslu and Girgin, 2010). However, Heidari et al. showed that the technicians' problem-solving skills improved after passing the problem-solving skills course (Heidari and Shahbazi, 2016). In addition, Chinar et al. reported that participants who rated their problem-solving skills better scored better on problem-solving skills, which is indicative of the effectiveness of self-confidence and self-awareness in this regard (Çinar et al., 2010). Considering the results of this study and the importance of empowering the EMS staff with problem-solving skills, it can be concluded that they have poor problem-solving skills. Therefore, to make the best decisions, providers must receive on-the-job training to strengthen problem-solving skills that can be used in this field and at the forefront of Iranian medical emergencies.

In this study, the participants made more use of adaptive strategies in emotion regulation, which was in line with the results of Habibi and Ebrahim's study (Ibrahim et al., 2020; Habibi Soola et al., 2022; Mirzaei et al., 2022). The results of these studies showed that emergency nurses and EMS staff were more likely to use positive coping strategies in dealing with stressful situations at work. However, as in our study, the participants were less likely to use negative strategies. It seems that in our study, due to repeated exposure to unpredictable and stressful situations, the participants were able to consider positive emotion regulation strategies as an adaptive strategy and change their emotional response to stressful events to improve their performance.

The results showed that refocusing on planning and positive reappraisal had the greatest effect on predicting problem-solving skills, which was consistent with the results of the Nasrolahi and Pour (2018) study. To our knowledge, no study has examined the relationship between problem-solving skills and emotion regulation in EMS technicians. It can be argued that executive actions imply the coherent use of several cognitive processes, and individuals use them to solve problems, plan, take action, organize, and monitor purposeful activities (Sam Daliri et al., 2017). Refocusing on planning and positive reappraisal in the problem-solving process are likely to increase the emergency technicians' confidence in their cognitive abilities. Perhaps this group of technicians is very flexible in coming up with strategies to cope with the primary control of stressors (planning and instrumental support) for the sake of secondary control (positive reappraisal) when coping with problems. The results of Betis and Lafuente's study showed that metacognitive self-regulation and executive performance skills (such as replanning and organizing) are important sources to improve students' abilities to make their strategies more flexible in addition to specific training aimed at enhancing their coping strategies (Bettis et al., 2017; De la Fuente et al., 2018). Azizi showed that the main problem for EMS personnel in dealing with accident scenes is psychological stress, with which rescuers deal in two ways: emotion-based management and problem-based approach. Therefore, emotional reactions such as intense emotions, emotional behavior, and emotional thoughts are manifested under emotion-based management (Azizi et al., 2021).

The results of multiple linear regression showed that acceptance and positive refocusing were negatively correlated with problem-solving skills among the EMS staff, which was in consistent with the results of a study by Kraaij and Garnefski (2015) and was not consistent with the results of Nasrallah's study (2018). Problem-solving skills are behaviors that one can rely on to overcome conflict, even during stressful missions (Cuijpers et al., 2018). For frontline health workers, a positive attitude toward a stressful situation is the main factor for protection against stress and is one of the key problem-solving methods (Babore et al., 2020). The results of a study showed that the most common problem-solving strategies used by healthcare providers were accepting a critical situation and using a positive attitude at work (Khalid et al., 2016). Mirzaei et al. reported that teaching life skills enhances adaptive strategies such as positive refocusing, and individuals act positively in consciously regulating their emotions and become more proficient in applying adaptive strategies such as positive refocusing (Mirzei and Hasani, 2015). Catastrophizing was negatively correlated with problem-solving skills among the EMS staff, which was consistent with the results of previous studies (Mirzei and Hasani, 2015; Nasrolahi and Pour, 2018; Bayram et al., 2022). The results of these studies show that increasing life skills reduces adaptive strategies such as catastrophizing. Moreover, Camberley et al. showed that increased catastrophizing elevate stress and anxiety and reduces people's performance and ability to deal with problems (Zlomke and Hahn, 2010). Emotional turmoil in the EMS technicians due to repeated exposure to emergencies and unpredictable events seems to be a reason for them to use catastrophizing solutions. This suggests that negative emotion regulation strategies in stressful situations are due to poor emotional skills and the inability to solve problems.

The results of multiple linear regression showed that passing the stress management course was one of the predictors of problem-solving skills in the participants. This result was consistent with the results of a study by Downey et al. (2011). Regarding the inverse correlation between problem-solving skills and passing the stress management course, it seems that most participants were not familiar with the stress management course and its effect on problem-solving skills in emergencies as only 25% of participants had passed this course. Adequate training is also a protective factor in the development of adverse psychiatric outcomes in medical respondents in all types of natural disasters (Azizi et al., 2021). The goal of the stress management course is to empower people to deal with stress, as a result of which they feel more comfortable and healthy and can cope better and more appropriately with stressful events. The results of multiple linear regression showed that delay and its reasons were predictors of problem-solving skills. Problem-solving skills were better in participants who stated they never had delays for missions than in those who had reasons for their delay. Time management and the absence of delays in missions can save technicians from confusion when the number of casualties is high (Bijani et al., 2021). Therefore, identifying the factors affecting the delay time and taking measures to reduce these factors can improve problem-solving and decision-making skills.

## Limitations

Since this study was performed on EMS personnel, it is impossible to generalize the results to other healthcare workers, so it is recommended to perform this study in other nursing departments. The small sample size was one of the main limitations of the study. At present, men are usually employed in pre-hospital emergency services in Iran. Hence, the majority of participants in the present study were male, and the results cannot be logically generalized to the female ones. Thus, it would be helpful to conduct a similar study on female technicians. Also, the use of a convenience sampling method and cross-sectional design based on the questionnaire is one of the main limitations of the present study that may affect the generalizability of the findings. Therefore, further research studies using random sampling and longitudinal design are recommended.

## Conclusion

Studies on problem-solving skills and emotion regulation were limited among the EMS personnel. The frontline healthcare staff are considered the most valuable assets in healthcare systems (Mirzaei et al., 2021). Based on the findings of this study and the significance of empowering EMS personnel in problem-solving skills through emotion regulation, it is possible to conclude that they have poor problem-solving skills. Therefore, to adopt the best coping measures against problems, the healthcare providers should receive in-service training to strengthen their problem-solving skills, which can be used in this field and at the forefront of Iranian medical emergencies. Moreover, today's knowledgeable and demanding society needs EMS technicians who can participate, solve problems, make decisions related to the profession, communicate, and have a mutual understanding. Accordingly, educators should also try to strengthen problem-solving skills, emotion regulation skills, and appropriate response times in a variety of ways. Effective problem-solving skills enable technicians to adapt to difficult and unpredictable situations.

## Data availability statement

The original contributions presented in the study are included in the article/supplementary material, further inquiries can be directed to the corresponding author/s.

## Ethics statement

The studies involving human participants were reviewed and approved by Research Committee of Ardabil University of Medical Sciences (Approval Number: IR.ARUMS.REC.1398.228). The patients/participants provided their written informed consent to participate in this study.

## Author contributions

Study design: MS, NM, and MM. Data collection: MS and AM. Data analysis: NM, MM, and AM. Manuscript preparation: AM, NM, MM, and MS. All authors read and approved the final manuscript.

## Funding

This work was supported by Ardabil University of Medical Science (arums-1336).

## Conflict of interest

The authors declare that the research was conducted in the absence of any commercial or financial relationships that could be construed as a potential conflict of interest.

## Publisher's note

All claims expressed in this article are solely those of the authors and do not necessarily represent those of their affiliated organizations, or those of the publisher, the editors and the reviewers. Any product that may be evaluated in this article, or claim that may be made by its manufacturer, is not guaranteed or endorsed by the publisher.
